# Alpha B-Crystallin Protects Rat Articular Chondrocytes against Casein Kinase II Inhibition-Induced Apoptosis

**DOI:** 10.1371/journal.pone.0166450

**Published:** 2016-11-16

**Authors:** Sung Won Lee, Jee Hyun Rho, Sang Yeob Lee, Seung Hee Yoo, Hye Young Kim, Won Tae Chung, Young Hyun Yoo

**Affiliations:** 1 Department of Rheumatology, Dong-A University College of Medicine, Busan, Republic of Korea; 2 Department of Anatomy and Cell Biology and Mitochondria Hub Regulation Center, Dong-A University College of Medicine, Busan, Republic of Korea; Pennsylvania State University, UNITED STATES

## Abstract

Although alpha (α)B-crystallin is expressed in articular chondrocytes, little is known about its role in these cells. Protein kinase casein kinase 2 (CK2) inhibition induces articular chondrocyte death. The present study examines whether αB-crystallin exerts anti-apoptotic activity in articular chondrocytes. Primary rat articular chondrocytes were isolated from knee joint slices. Cells were treated with CK2 inhibitors with or without αB-crystallin siRNA. To examine whether the silencing of αB-crystallin sensitizes rat articular chondrocytes to CK2 inhibition-induced apoptosis, we assessed apoptosis by performing viability assays, mitochondrial membrane potential measurements, flow cytometry, nuclear morphology observations, and western blot analysis. To investigate the mechanism by which αB-crystallin modulates the extent of CK2 inhibition-mediated chondrocyte death, we utilized confocal microscopy to observe the subcellular location of αB-crystallin and its phosphorylated forms and performed a co-immunoprecipitation assay to observe the interaction between αB-crystallin and CK2. Immunochemistry was employed to examine αB-crystallin expression in cartilage obtained from rats with experimentally induced osteoarthritis (OA). Our results demonstrated that silencing of αB-crystallin sensitized rat articular chondrocytes to CK2 inhibitor-induced apoptosis. Furthermore, CK2 inhibition modulated the expression and subcellular localization of αB-crystallin and its phosphorylated forms and dissociated αB-crystallin from CK2. The population of rat articular chondrocytes expressing αB-crystallin and its phosphorylated forms was reduced in an experimentally induced rat model of OA. In summary, αB-crystallin protects rat articular chondrocytes against CK2 inhibition-induced apoptosis. αB-crystallin may represent a suitable target for pharmacological interventions to prevent OA.

## Introduction

Osteoarthritis (OA) is the most common chronic joint disease in the elderly population. OA is a multifactorial disease with high morbidity that is characterized by degradation of the extracellular matrix and destruction of articular cartilage [1]. Chondrocytes are the only resident cells in human articular cartilage that are considered key players in the cartilage degeneration associated with OA [2,3]. This finding has led to the long-lasting assumption that cell death plays a central role in OA cartilage destruction. Signaling pathways involved in chondrocyte death have therefore been a focus of interest as pathogenic factors leading to joint cartilage degradation in OA.

Various cell death modes, such as apoptosis, necroptosis, autophagic cell death and mitotic catastrophe [4–8], have been observed in chondrocytes. Apoptosis has been extensively documented in chondrocyte death in the context of OA pathogenesis. Several stimuli, such as nitric oxide (NO) [9–12], prostaglandin E2 [13], Fas ligand [14, 15], tumor necrosis factor-α (TNF-α) [16], interleukin 1ß [17], and TRAIL [18], induce apoptosis in chondrocytes. The prevention of chondrocyte death has been suggested as a therapeutic strategy to limit cartilage damage.

Protein kinase casein kinase 2 (CK2), which is ubiquitously distributed in eukaryotic cells and acts as a messenger-independent protein serine/threonine kinase [19, 20], regulates apoptosis by phosphorylating various apoptosis-related factors [21–26]. CK2 inhibition induces articular chondrocyte death. Additionally, CK2 exerts protective effects against chondrocyte apoptosis through peroxynitrite-induced HO-1 expression [27]. Moreover, our previous studies have demonstrated that CK2 mediates anti-apoptotic effects during NO-induced apoptosis of rat articular chondrocytes [28] and that the down-regulation of CK2 facilitates TNF-α-mediated chondrocyte death through apoptosis and autophagy [4].

Crystallins are major structural proteins of the eye lens, where they occur as oligomers composed of two closely related subunits, alpha (α)A and αB [29, 30]. Both subunits are also expressed outside the vertebral eye lens [31, 32]. Entirely different non-lens roles for αB-crystallins have been assumed. αB–crystallin (heat-shock protein b5) belongs to the family of small heat-shock proteins (HSPs) and acts as a molecular chaperone induced by various stress stimuli, conferring cytoprotection by suppressing the aggregation of denatured proteins [33]. Accumulating evidence indicates that αB-crystallin negatively regulates apoptosis. Although previous studies demonstrated that αB-crystallin is expressed in human and bovine articular chondrocytes [34–36], little is known about the role of αB-crystallin in chondrocytes.

The present study was undertaken to examine whether αB-crystallin exerts anti-apoptotic activity in articular chondrocytes treated with CK2 inhibitors. As we demonstrate, αB-crystallin protects rat articular chondrocytes against CK2 inhibition-induced apoptosis.

## Materials and Methods

### Reagents

The following reagents were obtained commercially: rabbit polyclonal anti-human cytochrome c, rabbit polyclonal anti-human caspase-2L and goat polyclonal anti-human CKIIα antibodies from Santa Cruz Biotechnology (Santa Cruz, CA, USA); rabbit polyclonal anti-human αB-crystallin, phospho αB-crystallin-Ser19, -Ser45, -Ser59 and heat shock protein 90 (Hsp90) antibodies from Stressgen (Ann Arbor, MI, USA); a mouse monoclonal anti-human poly (ADP-ribose) polymerase (PARP) antibody from Oncogene (Cambridge, MA, USA); rabbit polyclonal anti-human caspase-3 and -7 and mouse monoclonal anti-human caspase-8 antibodies from Cell Signaling Technologies (Danvers, MA, USA); Avidin-biotin-peroxidase (ABC) complex, FITC-conjugated goat anti-rabbit and Texas Red-conjugated horse anti-mouse IgGs from Vector (Burlingame, CA, USA); HRP-conjugated donkey anti-rabbit and sheep anti-mouse IgGs from Amersham Pharmacia Biotech (Piscataway, NJ, USA); Dulbecco’s modified Eagle’s medium (DMEM) and fetal bovine serum (FBS) from Gibco BRL (Gaithersburg, MD, USA); TNF-α and ApopTag FITC In Situ Apoptosis Detection Kits from Millipore (Temecula, CA); mouse monoclonal anti-human SC35, α-tubulin and ß-actin antibodies, Hoechst 33342, dimethylsulfoxide (DMSO), RNase A, proteinase K, aprotinin, leupeptin, propidium iodide (PI), phenylmethylsulfonyl fluoride (PMSF), protein-A agarose, 5,6-dichlorobenzimidazol riboside (DRB), apigenin (API), 4,5,6,7-tetrabromobenzotriazole (TBB), mono-iodoacetate (MIA), 3,3’-diaminobenzidine (DAB) and type II collagenase from Sigma (St. Louis, MO, USA); caspase inhibitor I (zVAD-fmk) from Calbiochem (San Diego, CA, USA); 5,5’,6,6’-tetrachloro-1,1’,3,3’-tetraethylbenzimidazol carbocyanine iodide (JC-1) from Molecular Probes (Eugene, OR, USA); Lipofectamine® 2000 reagent from Invitrogen (Carlsbad, CA); and SuperSignal WestPico enhanced chemiluminescence western blotting detection reagent from Pierce (Rockford, IL, USA).

### Articular chondrocyte cell culture

As described previously [5], five-week-old male, specific pathogen-free Sprague Dawley rats were obtained from Samtoko (Osan, Korea). Rat articular chondrocytes for primary culture were isolated from knee joint cartilage slices via enzymatic digestion for 1 h with 0.2% type II collagenase (381 units/mg) in DMEM. After the isolated cells were collected by brief centrifugation, they were resuspended in DMEM supplemented with 10% (v/v) FBS, 50 mg/ml streptomycin and 50 units/ml penicillin (Gibco). The cells were plated on culture dishes at a density of 5–6 x 10^4^ cells/cm^2^. The medium was replaced every 2 days, and cells reached confluence after approximately 5 days in culture.

### Treatment with CK2 inhibitors

As described previously [4], 24 h after chondrocytes were subcultured, the original medium was removed. Cells were washed with PBS and then incubated in the same fresh medium. CK2 inhibitors (apigenin, DRB or TBB) from stock solutions were added to the medium to obtain 1–100 μM drug dilutions. Cells were harvested, stained with trypan blue and then counted using a hemacytometer. The concentration of PBS used in this study had no effect on chondrocyte proliferation in our preliminary studies.

### αB-crystallin siRNA

A 21-nucleotide RNA oligomer with 3’-dTdT overhangs was synthesized by Dharmacon Research (Thermo Fisher Scientific, Lafayette, CO, USA) through the “ready-to-use” option. The AA-N19 mRNA targets an αB-crystallin target sequence (5’-AAUUGACCAGUUCUUCGGAGA-3’). As a negative control, the same nucleotides were scrambled to form a non-genomic oligomer.

### CK2 siRNA

Rat CK2 siRNA (SMART pool; L-096197-02-0020) was purchased from Thermo Scientific (Hudson, NH, USA). As a negative control, the same nucleotides were scrambled to form nongenomic oligomer.

### αB-crystallin siRNA transfection or combination treatment with CK2 inhibitors

siRNA transfection was performed using siPORT Amine and Opti-MEM media. Cells grown to a confluence of 40% to 50% in 6-well plates were transfected with siRNA at a final concentration of 100 nM per well. The transfection mixture was added to each well and incubated for 4 h. Then, 2 ml of growth medium was added, and cells were incubated for another 20 h. After the siRNA transfection medium was removed, each well was washed with PBS solution. Cells were treated with various concentrations of CK2 inhibitors for 24 h.

### Overexpression of αB-crystallin

Plasmid pcDNA3 rat-αBC was provided by Dr. J-K. Lee (Inha University, Incheon, South Korea). Chondrocyte were seeded in six-well plates (10^5^ cells/well) and were transiently transfected with each construct using Lipofectamine 2000, according to the manufacturer's instructions. Briefly, 2μg of plasmid DNA was mixed with 6μl of Lipofectamine 2000 and then incubated with Opti-MEM. The plasmid DNA–Lipofectamine 2000 complex was added to the cells, and the mixture was further incubated for 4 h at 37°C. After incubation, the medium was replaced with 2 ml of growth medium, and the cells were maintained for an additional 20 h. Twenty-four hours after transfection, the plasmid DNA transfection medium was removed, and each well was washed with PBS solution. Cells were further exposed new fresh medium for 24 h. V, cells tranfected with pcDNA3 vector.

### Cell viability assay

As described previously [5], cell viability was determined with a Vi-Cell (Beckman Coulter, CA, USA) cell counter, which performs an automated trypan blue exclusion assay.

### Nuclear morphology study to assess apoptosis

As described previously [5], cell suspensions were cytospun onto clean fat-free glass slides using a cytocentrifuge. Centrifuged samples were fixed in 4% paraformaldehyde for 10 min and stained with 10 μg/ml PI or in 4 μg/ml Hoechst 33342 for 30 min at 4°C. Cells were observed and imaged with an epifluorescence microscope.

### Quantification of DNA hypoploidy and cell cycle phase analysis by flow cytometry

As described previously [4], ice-cold 95% ethanol with 0.5% Tween-20 was added to cell suspensions to a final concentration of 70% ethanol. Fixed cells were pelleted and washed in 1% BSA-PBS solution. Cells were re-suspended in 1 ml of PBS containing 11 Kunitz U/ml RNase, incubated at 4°C for 30 min, washed once with BSA-PBS, and re-suspended in PI solution (50 μg/ml). After the cells were incubated at 4°C for 30 min in the dark and washed with PBS, DNA content was measured on an Epics XL flow cytometer (Beckman Coulter, FL, USA), and data were analyzed using Multicycle software, which allows the simultaneous estimation of cell cycle parameters and apoptosis.

### Flow cytometric analysis of Annexin V-FITC binding for apoptosis

As described previously [4], apoptosis was determined by flow cytometry with an Annexin V-FITC apoptosis detection kit according to the manufacturer’s instruction. The cells (1 × 10^6^) were washed twice with ice-cold PBS, resuspended in 500 μl of Annexin V binding buffer and incubated with 5 μl of Annexin V-FITC and 10 μl of PI. The cells were incubated for 10 min at room temperature in the dark. After double staining, the cells were analyzed using an Epics XL flow cytometer.

### Western blot analysis

As described previously [4], equal amounts of protein were separated by 7.5–15% sodium dodecyl sulfate-polyacrylamide gel electrophoresis (SDS-PAGE). The gels were transferred to nitrocellulose membranes (Amersham Pharmacia Biotech, Piscataway, NJ, USA) and reacted with antibodies. Immunostaining was analyzed by incubating the membranes with SuperSignal WestPico enhanced chemiluminescence substrate and detecting with LAS-3000 Plus.

### Immunofluorescence staining, confocal microscopy and quantification

As described previously [5], cells cultured on coverslips were incubated with a primary antibody for 1 h at 37°C, washed 3 times for 5 min each with PBS, incubated with a FITC-conjugated secondary antibody for 1 h at room temperature and counterstained with propidium iodide. For double immunofluorescent staining, cells were incubated with two different primary antibodies for 1 h at 37°C and washed three times for 5 min each with PBS. FITC-conjugated and Texas Red-conjugated secondary antibodies were used. Fluorescence images were observed and analyzed using a Zeiss LSM 510 laser-scanning confocal microscope (Goettingen, Germany).

### Mitochondrial membrane potential (MMP) assay

As described previously [5], MMP disruption was measured using a specific fluorescent probe, JC-1, added directly to the cell culture medium (5 μg/ml final concentration) and incubated for 15 min at 37°C. Flow cytometry was used to measure JC-1 signals (Epics XL; Beckman Coulter). Data were acquired and analyzed using EXPO32 ADC XL 4-color software.

### TUNEL staining of cell suspensions

Cell suspensions were cytospun onto clean fat-free glass slides in a cytocentrifuge. After fixation with 4% paraformaldehyde, the cells were incubated with the terminal deoxynucleotidyl transferase (TdT) enzyme for 1 h at 37°C, and antidigoxigenin-FITC was applied for 30 min at room temperature. After the addition of PI Antifade Mounting Medium, fluorescent images were obtained and analyzed using a Zeiss LSM 510 laser-scanning confocal microscope.

### Co-immunoprecipitation (Co-IP)

As described previously [5], cell extracts incubated with antibodies were precipitated with protein A-agarose beads. Immunoprecipitated proteins or aliquots were separated by SDS-PAGE, and western blot analysis was performed as described. Each co-IP experiment was confirmed via reciprocal IP (data not shown).

### Experimental OA in rats

Twelve-week-old male Sprague-Dawley rats weighing 200–250 gm were used. Rats were housed under ad libitum food and water conditions. The anterior cruciate ligament in the right knee joint was transected using a cutting hook. Animals were maintained within their cages, where they moved freely without post-operative casts. Starting three days after the operation, the rats were trained in a treadmill exercise box for 10 min every hour between 9 am and 5 pm over a 4-week period. The right knee joints from six rats subjected to surgery were used as the experimental group. The right knees of an additional 6 rats that were not subjected to surgery were used as controls. The study was approved by the ethics committee of Dong-A University and fulfilled the guidelines for animal experiments established by The Korean Academy of Medical Sciences.

### Tissue preparation and immunohistochemical analysis

At the end of the 4-week training period, animals were sacrificed by ether inhalation. The right knee joint was dissected from each animal, and tibial condyles were fixed in PBS (pH 7.4) containing 4% paraformaldehyde, decalcified in 12.5% EDTA, dehydrated, and embedded in paraffin blocks. Five-micrometer microsections were incubated in goat serum solution diluted 1:70 for 30 min at room temperature and then with primary antibody diluted 1:100 for 2 h at room temperature. Next, sections were incubated with secondary antibody for 1 h at 37°C and developed using the ABC complex. Peroxidase staining was revealed with DAB and examined by light microscopy. Histological images were obtained and analyzed using an Aperio ScanScope® CS system. Total numbers of cells positive for αB-crystallin and its phosphorylated forms and CK2 in four fields per animal were counted by an observer blinded to the experiment, and the percentage of positive cells was calculated.

### Statistical analysis

The six independent experiments were carried out in triplicate. All data are displayed as the mean ± standard deviation (SD). Normality was verified with the Shapiro-Wilk test, and homogeneity of variances was verified using Levene's Test prior to statistical analysis. One-way analysis of variance (ANOVA) followed by Scheffe's post hoc test was used to analyze differences in chondrocyte viability under each treatment condition (according to concentration or treatment). Additionally, linear contrast analysis was performed to evaluate the linear trend between the viability means based on each treatment condition. For cases that violated the assumptions of normality and homogeneity of variance, a Kruskal-Wallis test followed by the Mann-Whitney U test were performed to analyze viability differences within groups. The results obtained from the experimental OA rats and control groups were tested for statistical significance with the Kruskal-Wallis nonparametric test. Resulting two-tailed P-values < 0.05 were regarded as statistically significant. SPSS version 21.0 (IBM Corp, Armonk, NY, USA) was used to perform statistical analyses.

## Results

### αB-crystallin knockdown renders rat articular chondrocytes vulnerable to subtoxic doses of DRB

**Fig 1:**
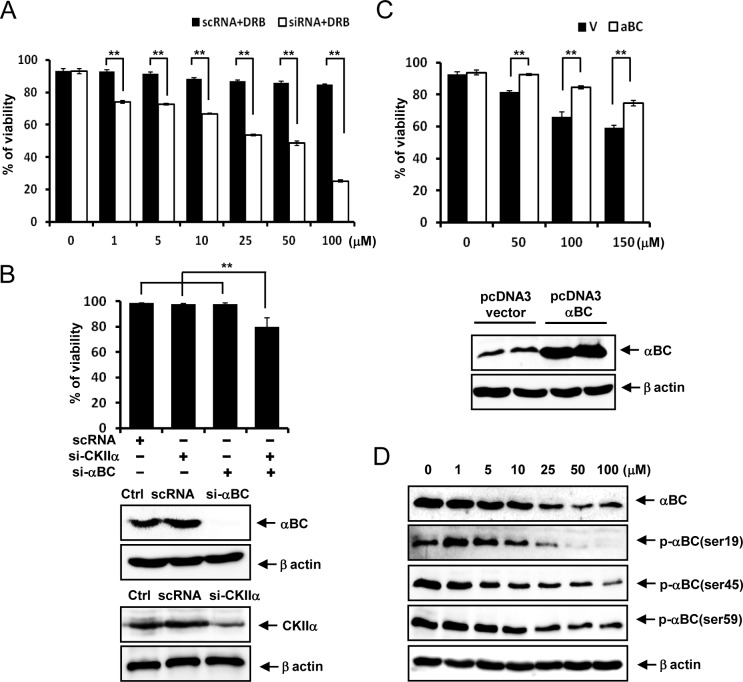
DRB modulates the phosphorylation and subcellular localization of αB-crystallin. (A) αB-crystallin knockdown rendered rat articular chondrocytes vulnerable to subtoxic doses of DRB. 24 h after DRB treatment. (B) Knockdown of both αB-crystallin and CK2 genes reduced the viability of rat articular chondrocytes. (C) Overexpression of αB-crystallin reversed the reduced viability of rat articular chondrocytes to toxic doses of DRB. 72 h after DRB treatment. (D) DRB reduced αB-crystallin protein expression and P-αB-crystallin-Ser-19, Ser-45 and Ser-59 levels. Each experiment, six in total, was conducted with three animals each. In each experiment, the cells from three animals were pooled and analyzed in triplicate. Values are the mean ± SD. * *P* < 0.05, ** *P* <0.01 vs. control.

Whereas the CK2 inhibitor DRB at doses ranging from 1 to 100 μM did not significantly reduce the viability of primary cultured rat articular chondrocytes, αB-crystallin knockdown rendered rat articular chondrocytes vulnerable to these subtoxic doses of DRB (Fig 1A). While αB-crystallin or CK2 knockdown did not reduce the viability of primary cultured rat articular chondrocytes, knockdown of both genes slightly reduced their viability (Fig 1B). Overexpression of αB-crystallin reversed the reduced viability of rat articular chondrocytes to toxic doses of DRB (Fig 1C). We next examined whether DRB modulated αB-crystallin expression levels and phosphorylation status. DRB reduced αB-crystallin protein expression as well as the levels of P-αB-crystallin-Ser-19, Ser-45 and Ser-59 (Fig 1D).

### DRB modulates the phosphorylation and subcellular localization of αB-crystallin in rat articular chondrocytes

**Fig 2:**
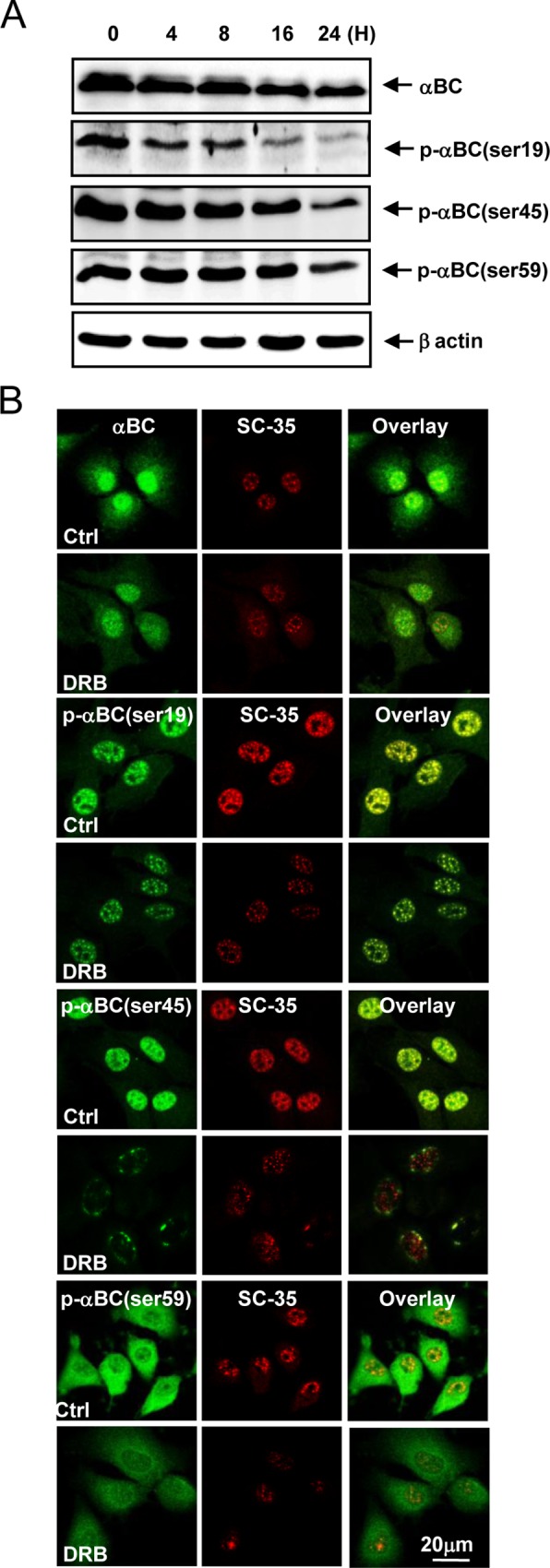
DRB modulates the phosphorylation and subcellular localization of αB-crystallin. (A) DRB treatment alone for various durations reduced αB-crystallin protein expression and P-αB-crystallin-Ser-19, Ser-45 and Ser-59 levels in a time-dependent manner. (B) DRB treatment induced the partial loss of cytoplasmic and nuclear αB-crystallin, whereas P-αB-crystallin-Ser19 and P-αB-crystallin-Ser45 localization within SC35 speckles was sustained. Each experiment, six in total, was conducted with three animals each. In each experiment, the cells from three animals were pooled and analyzed in triplicate.

**Fig 3:**
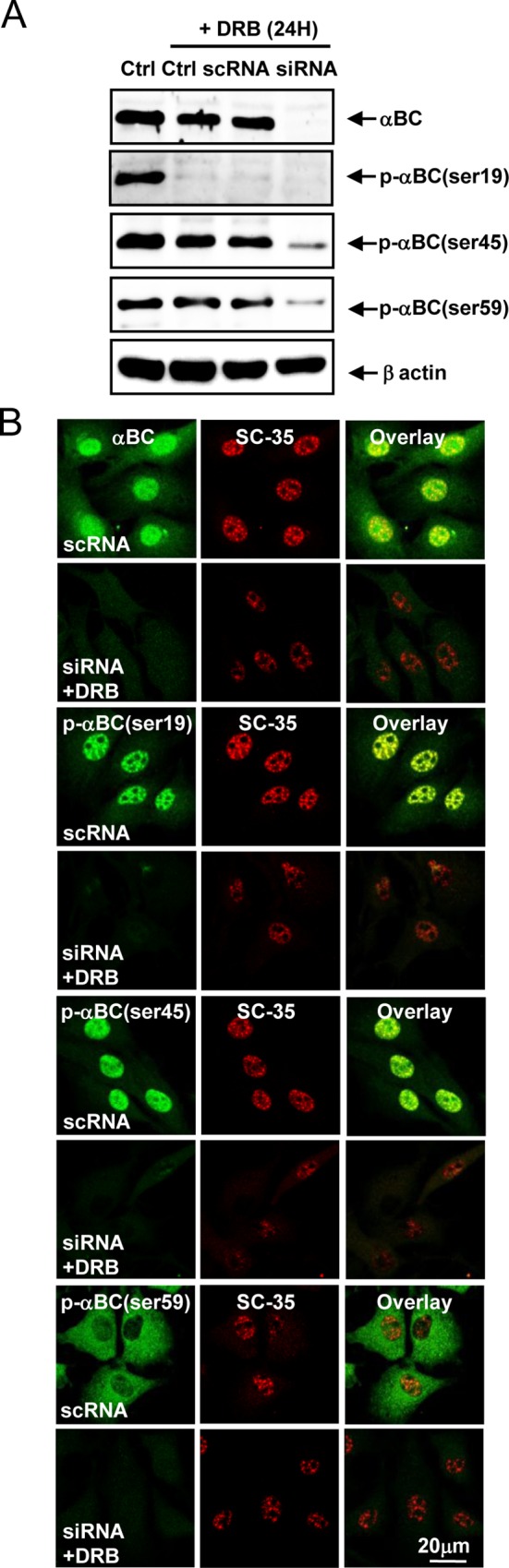
Silencing of αB-crystallin markedly suppressed the expression of αB-crystallin and resulted in the disappearance of P-αB-crystallin-Ser-19 and Ser-45 from SC35 speckles. (A) Silencing of αB-crystallin markedly suppressed αB-crystallin expression. (B) Silencing of αB-crystallin resulted in the notable disappearance of P-αB-crystallin-Ser-19 and Ser-45 from SC35 speckles. Each experiment, six in total, was conducted with three animals each. In each experiment, the cells from three animals were pooled and analyzed in triplicate.

Because the viability of rat articular chondrocytes treated with 50 μM DRB and αB-crystallin siRNA was approximately 50% of control values, this combination treatment was used in all subsequent studies. Treatment with 50 μM DRB alone for various durations decreased both αB-crystallin protein expression and levels of P-αB-crystallin-Ser-19, Ser-45 and Ser-59 in a time-dependent fashion (Fig 2A). In untreated control articular chondrocytes, αB-crystallin was distributed in both the cytoplasm and nuclei. DRB treatment induced the partial loss of cytoplasmic and nuclear αB-crystallin. Importantly, the localization of P-αB-crystallin-Ser19 and P-αB-crystallin-Ser45 within SC35 speckles was sustained in chondrocytes treated with DRB. P-αB-crystallin-Ser-59, which was primarily found in the cytoplasm of untreated control rat articular chondrocytes, was markedly decreased in chondrocytes treated with DRB (Fig 2B). Silencing of αB-crystallin markedly suppressed the expression of αB-crystallin protein as well as P-αB-crystallin-Ser-19, Ser-45 and Ser-59 expression (Fig 3A). Silencing of αB-crystallin resulted in the marked disappearance of P-αB-crystallin-Ser19 and P-αB-crystallin-Ser45 from SC35 speckles (Fig 3B).

### Silencing of the αB-crystallin gene sensitizes rat articular chondrocytes to DRB-induced apoptosis

**Fig 4:**
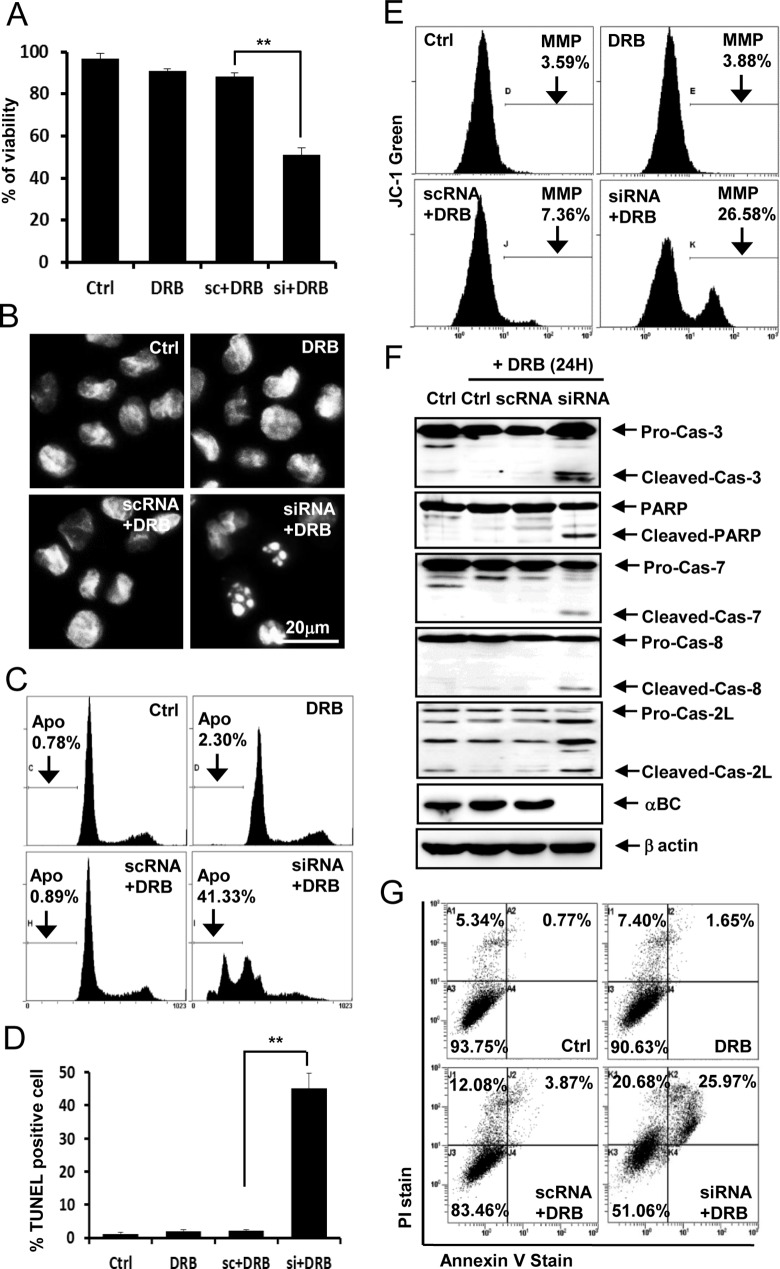
Silencing of the αB-crystallin gene sensitizes rat articular chondrocytes to DRB-induced apoptosis. (A) Silencing of the αB-crystallin gene significantly sensitized rat articular chondrocytes to DRB-induced cell death. (B) DRB administered in combination with si-αB-crystallin induced nuclear condensation and fragmentation. (C) Subdiploid apoptotic cells (Apo) accumulated among chondrocytes treated with DRB and si-αB-crystallin. (D) DRB administered in combination with si-αB-crystallin increased the percentage of TUNEL-positive cells. (E) DRB administered with si-αB-crystallin reduced MMP levels in rat articular chondrocytes. (F) DRB administered in combination with si-αB-crystallin produced caspase-3, -7, -8, and -2L and PARP cleavage products. (G) Flow cytometry demonstrated that 50 μM DRB administered in combination with si-αB-crystallin increased the population of cells undergoing apoptosis. Representative flow cytometry data based on Annexin V-FITC/PI double staining are shown. Each experiment, six in total, was conducted with three animals each. In each experiment, the cells from three animals were pooled and analyzed in triplicate. Values are the mean ± SD. * *P* < 0.05, ** *P* <0.01 vs. control.

To examine the mechanism by which αB-crystallin gene silencing sensitizes rat articular chondrocytes to DRB-induced cell death, apoptosis was analyzed in various ways. Viability assays (Fig 4A), evaluation of nuclear morphology (Fig 4B), quantification of DNA hypoploidy by flow cytometry (Fig 4C), TUNEL staining and quantification (Fig 4D), measurement of MMP (Fig 4E), western blot analysis of caspase subtypes (Fig 4F), and a flow cytometry-based Annexin V staining assay (Fig 4G) indicated that αB-crystallin gene silencing induces mitochondria- and caspase-dependent apoptosis in rat articular chondrocytes treated with DRB.

### DRB treatment dissociates αB-crystallin from CK2

**Fig 5:**
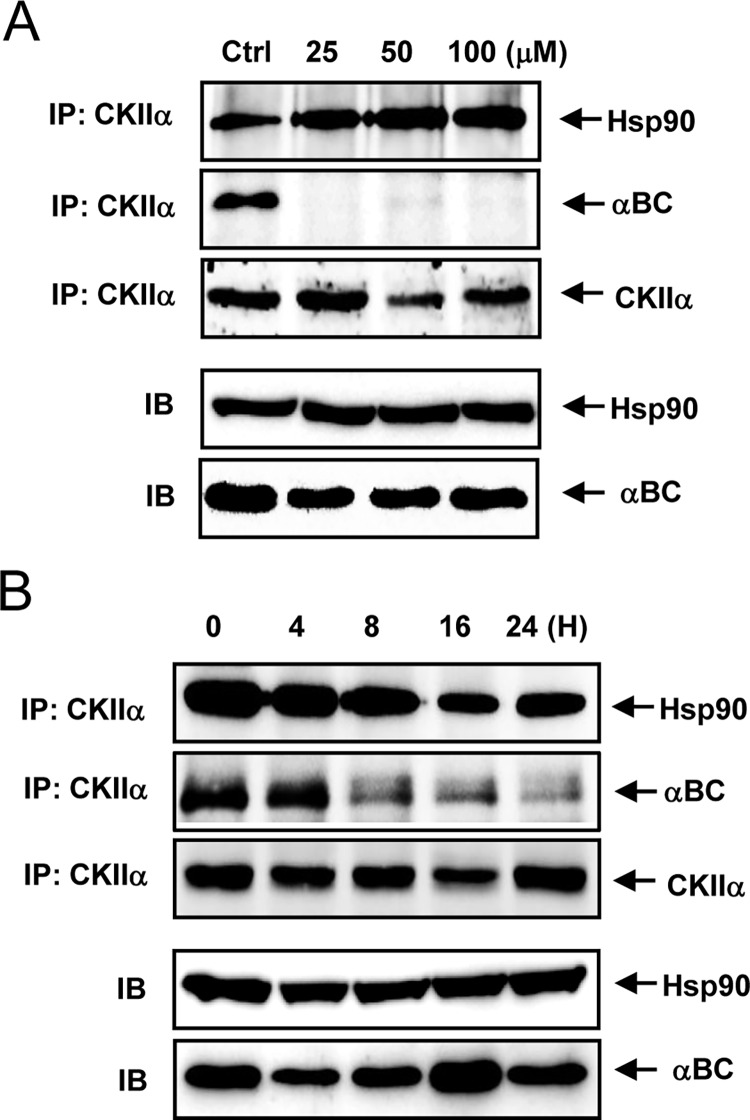
DRB treatment dissociates αB-crystallin from CK2. (A) DRB treatment for 24 h caused αB-crystallin to dissociate from CK2. (B) DRB treatment caused αB-crystallin to dissociate from CK2 in a time-dependent manner. Each experiment, six in total, was conducted with three animals each. In each experiment, the cells from three animals were pooled and analyzed in triplicate.

We next examined whether DRB reduces the association between CK2 and αB-crystallin. Although DRB did not reduce the association between CK2 and HSP90, DRB treatment markedly reduced the interaction between CK2 and αB-crystallin in rat articular chondrocytes (Fig 5A and 5B).

### Two additional CK2 inhibitors, apigenin (API) and 4,5,6,7-tetrabromobenzotriazole (TBB), induce similar phenotypic changes in rat articular chondrocytes

**Fig 6:**
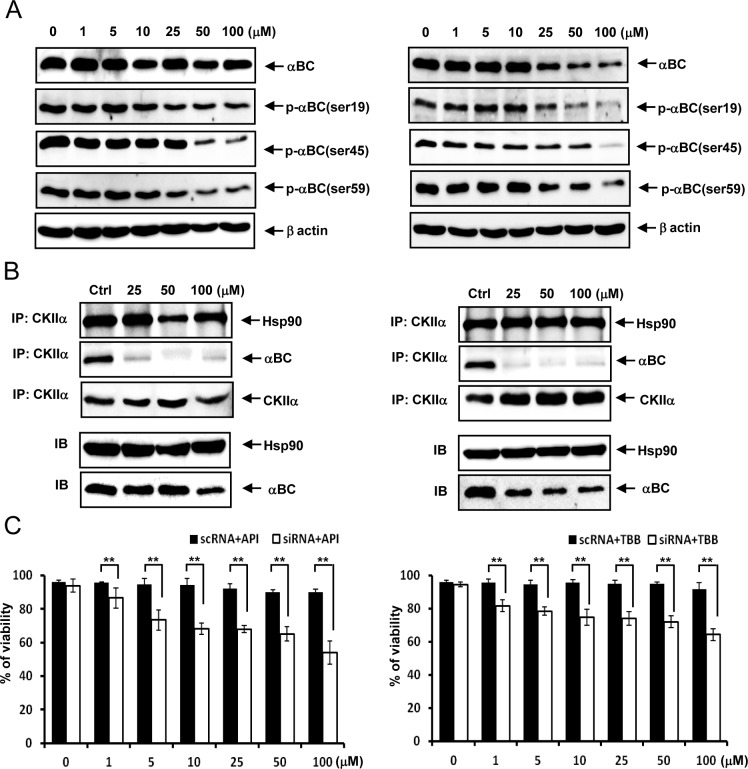
API and TBB exhibit similar phenotypic changes in rat articular chondrocytes. (A) Both API and TBB reduced the expression of αB-crystallin protein and the levels of P-αB-crystallin-Ser-19, Ser-45 and Ser-59 in rat articular chondrocytes. (B) Both API and TBB markedly reduced the interaction between CK2 and αB-crystallin in rat articular chondrocytes. (C) Silencing of the αB-crystallin gene significantly sensitized rat articular chondrocytes to API- and TBB-induced cell death. Each experiment, six in total, was conducted with three animals each. In each experiment, the cells from three animals were pooled and analyzed in triplicate. Values are the mean ± SD. * *P* < 0.05, ** *P* <0.01 vs. control.

Both API and TBB not only reduced αB-crystallin protein expression levels and P-αB-crystallin-Ser-19, Ser-45 and Ser-59 levels in rat articular chondrocytes (Fig 6A) but also markedly reduced the interaction between CK2 and αB-crystallin in rat articular chondrocytes (Fig 6B). We observed that αB-crystallin protects rat articular chondrocytes from API- or TBB-induced cell death (Fig 6C).

### The population of rat articular chondrocytes expressing CK2 and αB-crystallin is reduced in an experimentally induced rat model of OA

**Fig 7:**
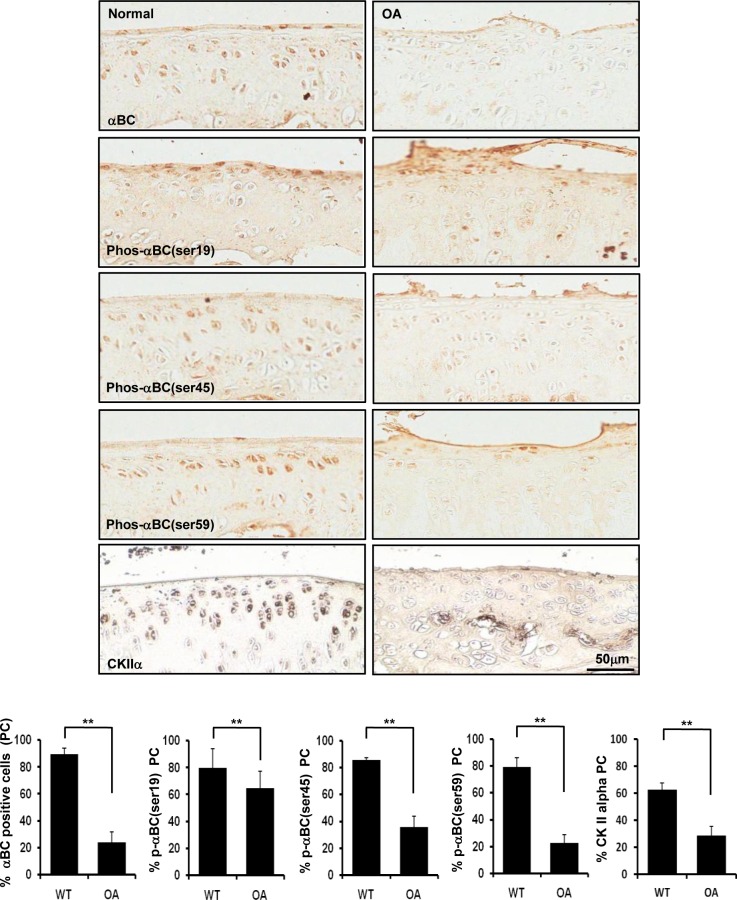
The population of rat articular chondrocytes expressing αB-crystallin and its phosphorylated forms and CK2 is reduced in an experimentally induced osteoarthritis rat model. Representative immunostaining of rat articular cartilage from a surgically induced OA model (n = 6 per group). Values are the mean ± SD. * *P* < 0.05, ** *P* <0.01 vs. control.

We examined whether αB-crystallin expression is decreased in rat articular chondrocytes in an experimentally induced rat OA model. Importantly, the population of rat articular chondrocytes expressing αB-crystallin and P-αB-crystallin-Ser-19, Ser-45 and Ser-59 was significantly reduced in an experimentally induced rat model of OA. We observed that the population of rat articular chondrocytes expressing CK2 was also significantly reduced in an experimentally induced rat model OA (Fig 7).

## Discussion

Previous studies demonstrated that αB-crystallin is expressed in chondrocytes [34–36]. Furthermore, αB-crystallin expression is induced during the early stages of differentiation of the chondroprogenitor cell line ATDC-5, and αB-crystallin is constitutively expressed in cultured human and bovine articular chondrocytes. However, the role of αB-crystallin in OA-affected chondrocytes is not well understood. A study revealed the differential transcription of αB-crystallin at the mRNA level and the differential abundance of αB-crystallin protein in OA-affected articular chondrocytes compared with healthy chondrocytes, indicating that αB-crystallin is a newly identified potential regulator of matrix gene expression. Additionally, the association between reduced αB-crystallin levels and reduced levels of aggrecan, type II collagen, and bone morphogenetic protein 2 demonstrates that αB-crystallin is involved in the phenotypic changes exhibited by chondrocytes during OA development [36]. However, to date, no reports have indicated that αB-crystallin plays a role in chondrocyte death pertaining to OA pathogenesis. In the present study, silencing of the αB-crystallin gene induced apoptosis in rat articular chondrocytes treated with CK2 inhibitors, and the population of rat articular chondrocytes expressing αB-crystallin was reduced in an experimentally induced OA rat model. Thus, αB-crystallin protects articular chondrocytes against apoptosis and prevents, at least in part, the onset of OA.

Numerous studies have reported the molecular mechanisms underlying the anti-apoptotic actions exerted by αB-crystallin. αB-crystallin inhibits the autocatalytic maturation of caspase-3 [37], the translocation of Bax and Bcl-2 from the cytosol to the mitochondria [38], caspase-3 and PARP (poly [ADP-ribose] polymerase) activation [39] and the translocation of p53 into mitochondria [40]. αB-crystallin also decreases apoptosis by preventing RAS activation and inhibiting the RAF/MEK/ERK pathway [41]. We previously demonstrated that the dissociation of caspase-7 and -3 from αB-crystallin correlates with apoptotic induction, indicating that αB-crystallin inhibits caspase activation by physically interacting with caspase subtypes not only in the cytoplasm but also in the nucleus [42]. Furthermore, αB-crystallin expressed in glioma cells incapacitates the anti-apoptotic activity exerted by XIAP [43].

αB-crystallin is distributed both in the cytoplasm and nuclei. In the nucleus, αB-crystallin forms nuclear body–like structures and localizes to SC35 speckles. αB-crystallin phosphorylation, which primarily occurs at serines 19, 45 and 59, determines its subcellular localization. Furthermore, the phosphorylation of αB-crystallin modulates its properties [44,45]: whereas Ser59 phosphorylation is required for nuclear import, Ser45 phosphorylation is crucial for speckle localization [46]. Ser45 and Ser59 phosphorylation of αB-crystallin plays a pivotal role in αB-crystallin-mediated cytoprotection [47]. Additionally, phosphorylated αB-crystallin sustains its association with caspase-3 and -7 in SC35 speckles, playing a pivotal role in preventing apoptosis [42].

In the present study, we demonstrated that the localization of P-αB-crystallin-Ser19 and P-αB-crystallin-Ser45 within SC35 speckles was sustained in articular chondrocytes treated with DRB alone. In contrast, silencing of the αB-crystallin gene completely suppressed αB-crystallin location within SC35 speckles. While DRB treatment alone did not reduce viability, DRB in conjunction with αB-crystallin siRNA markedly reduced viability. Thus, the sustenance of P-αB-crystallin-Ser19 and P-αB-crystallin-Ser45 within SC35 speckles plays a role in protecting chondrocytes against DRB-stimulated apoptosis. We assume that the association between caspase subtypes and P-αB-crystallin-Ser19 and P-αB-crystallin-Ser45 in SC35 speckles represents a last line of defense to resist DRB-induced apoptosis.

Additionally, two additional CK2 inhibitors, apigenin and TBB, induced phenotypic changes similar to DRB; therefore, CK2 inhibition renders chondrocytes vulnerable to apoptosis by inhibiting αB-crystallin action. A previous study reported that HSP90 binds and protects CK2 from self-aggregation and enhances its kinase activity in a mouse fibroblast L cell line [48]. In this study, DRB treatment markedly reduced the interaction between CK2 and αB-crystallin in rat articular chondrocytes. Although further studies are required, our data suggest that the interaction between αB-crystallin and CK2 plays a role in inducing the anti-apoptotic activity of CK2 in rat chondrocytes.

Furthermore, αB-crystallin expression was decreased in rat articular chondrocytes in an experimentally induced rat OA model. Thus, decreased αB-crystallin expression, at least in part, may be implicated in the induction of OA in our experimental model. However, numerous other factors in addition to the downregulation of CK2 may also be involved. It is not clear whether the mechanism described here accurately reflects the protective effects exerted by αB-crystallin against CK2 inhibition-induced chondrocytes apoptosis; therefore, the exact mechanism by which this process is regulated by αB-crystallin warrants further investigation.

In conclusion, αB-crystallin is an anti-apoptotic molecule that confers protection upon stressed chondrocytes and represents a rational target for the development of new drugs to treat OA.
